# Soil disinfestation and optimized nutrient management reduces nitrogen leaching and shapes soil microbial community composition in greenhouse cucumber production systems

**DOI:** 10.3389/fmicb.2025.1663041

**Published:** 2025-09-05

**Authors:** Xilin Guan, Bin Liu, Wenfan Bian, Yufeng Zhang, Xinhao Gao, Xinping Chen, Yan Li, Shenzhong Tian

**Affiliations:** ^1^State Key Laboratory of Nutrient Use and Management, Key Laboratory of Wastes Matrix Utilization, Ministry of Agriculture and Rural Affairs, Institute of Agricultural Resources and Environment, Shandong Academy of Agricultural Sciences, Jinan, China; ^2^Interdisciplinary Research Center for Agriculture Green Development in the Yangtze River Basin, College of Resources and Environmental Sciences, Southwest University, Chongqing, China; ^3^Key Lab of Plant-Soil Interaction, MOE, College of Resources and Environmental Sciences, China Agricultural University, Beijing, China

**Keywords:** greenhouse cucumber, bio-organic fertilizer, nutrient accumulation, microorganisms, dissolved inorganic nitrogen

## Abstract

High nutrient input leads to problems such as excessive accumulation of soil nutrients, and imbalance of microbial communities. However, there remains significant gaps in the impact of soil disinfestation and optimized nutrient management measures on the growth and soil conditions of greenhouse cucumber. This study investigated the effects of non-disinfected soil with conventional nutrient treatment (CK), after disinfection with conventional chicken manure application (FP), after disinfection with bio-organic fertilizer application (BF), and after disinfection with organic materials application (OM) on cucumber growth performance, soil nutrients, and microbial communities. The results indicated that compared to the CK, FP, BF, and OM could significantly increase cucumber yield, dry weight, nitrogen uptake, and the nutritional yield of potassium, calcium, and magnesium, with BF showing the most pronounced effect. BF effectively alleviated the migration of dissolved inorganic nitrogen in the 0–200 cm soil layer, thereby reducing its loss. In addition, BF reduced the accumulation of nutrients such as total nitrogen, available phosphorus, available potassium, and available magnesium in the 0–20 cm soil layer. The optimization measures altered the species diversity of the soil microbial community. In the BF group, microorganisms showed a negative correlation with soil properties, while OM was positively correlated with pH and C/N, with *norank_c__Subgroup_6* and *Gaiella* being the dominant bacteria. Functional analysis revealed that carbon and nitrogen metabolism functions were significantly enriched in BF-associated bacteria compared to all other groups. These findings provide new insights and strategies for saving resources, improving crop quality, and reducing nutrient accumulation and waste.

## 1 Introduction

Soil is the material foundation essential for human survival, and soil microorganisms are key driving forces in nutrient cycling, organic matter transformation, and the enhancement of crop yield and productivity. Therefore, microbial activity, abundance, and community composition directly influence soil health and quality, and largely determine the sustainable productivity of agricultural land (Ahammed et al., 2025; Taheri et al., 2025). However, in greenhouse vegetable production systems, long-term irrational nutrient inputs and continuous monoculture often lead to an increase in the abundance of harmful microorganisms in the soil, while the relative abundance of beneficial microbes declines, thereby contributing to the occurrence of soil-borne diseases in agricultural production (Su et al., 2024; Wang et al., 2024).

At present, vegetable production commonly employs soil disinfectants (Son et al., 2023) or practices such as crop rotation and variety replacement (Dou et al., 2025; Kim et al., 2025; Ma et al., 2025) to suppress the occurrence of soil-borne diseases. However, some disinfectants may leave chemical residues in the soil that negatively affect crop growth. Meanwhile, crop rotation in greenhouse vegetable systems is often constrained by technical and management limitations. Previous studies have demonstrated that soil disinfection can promote plant growth. The recovery of microbial communities after sterilization depends on the presence of living plants, and the final microbial community structure varies according to crop species. In crops grown in disinfected soils, disinfection has been shown to enhance the functions of common plant-beneficial microorganisms (Li et al., 2019). For example, physical and chemical properties as well as microbial communities of tobacco inter-roots were deteriorated when infected with mosaic virus, a phenomenon that is more pronounced in unsterilized soils (Liu et al., 2024). Therefore, for crops cultivated in greenhouses, utilizing natural conditions such as greenhouse solarization (high-temperature sealing of the greenhouse) as a sterilization method can reduce resource consumption, help reestablish a balanced microbial community, and create a favorable soil environment for plant establishment and growth.

Optimizing nutrient management to meet crop nutritional requirements is a fundamental step toward improving vegetable yield and quality while minimizing environmental pollution. Previous studies have reported various nutrient management strategies that influence vegetable production and quality, including fertilizer reduction, coordinated water supply, application of enhanced-efficiency fertilizers, substitution of chemical fertilizers with organic amendments, and crop mulching. However, excessive nutrient input can lead to leaching losses, posing threats to environmental safety. For instance, in wheat cultivation, optimized water and nitrogen management has been shown to enhance resource use efficiency without compromising yield. The application of biochar has improved nutrient availability and reduced yield loss caused by irrigation deficits (Zhang et al., 2024). Under controlled nutrient management, altering microbial communities in nutrient solutions has been used to improve fertilizer use efficiency and yield in greenhouse lettuce (Zhao et al., 2025). Controlled-release phosphorus fertilizers have achieved the highest microbial diversity and the greatest relative abundance of beneficial microbes, while enhancing soil enzyme activity, soil fertility, and soybean growth (Qu Z. et al., 2025). The combined application of farmyard manure and bio-inoculants has improved microbial diversity, abundance, and resilience, contributing to sustainable crop production (Datta et al., 2025). Seguel and colleagues developed a biochar-based controlled-release fertilizer that replaced conventional fertilizers, thereby improving nutrient use efficiency, enhancing grain productivity, and reducing nutrient loss (Meier et al., 2025). However, the application and potential of optimized nutrient management in greenhouse cucumber production remain largely unknown.

The application of bio-organic fertilizers is one of the important approaches to improving soil nutrient status and health. By combining soil sterilization measures with the application of bio-organic fertilizers, antagonistic microorganisms can be artificially introduced to establish a disease-suppressive soil microbial community, thereby reducing the use of agricultural chemicals and enhancing soil health. The application of bio-organic fertilizer has been shown to not only increase the fresh root weight and active compound content of *Scutellaria baicalensis*, but also to improve the composition of the rhizosphere fungal community (Guo et al., 2025). In potato cultivation, optimized organic fertilization alleviated early-stage wilt disease, slowed yield reduction, and increased the content of particulate organic matter (Qu X. et al., 2025). The combined application of biochar and microbial organic fertilizer improved soil fertility by 161% and increased the abundance of antagonistic Chaetomiaceae fungi (He et al., 2025). The combined use of bio-organic fertilizer and soil amendments reduced soil salinity, enhanced enzyme activity, improved saline-alkaline soils, and significantly increased rice yield (Xiao et al., 2025). The substitution of part of the chemical fertilizer with bio-organic fertilizer not only reduced resource consumption, but also enhanced the abundance of dominant bacterial and fungal groups and increased the yield of *Brassica chinensis* (Jin et al., 2022). Bio-organic fertilizers have shown great potential in enhancing crop yield, improving soil health, and enriching beneficial microbial communities. Therefore, it is necessary to compare the effects of greenhouse solarization with calcium cyanamide and the combined application of traditional manure, bio-organic fertilizer, and organic materials on cucumber yield, soil fertility, and microbial characteristics in greenhouse systems.

This study focused on greenhouse-grown cucumber in Lanling County, with the following main objectives: (1) To investigate the effects of nutrient management practices on cucumber growth performance, (2) to quantitatively analyze the migration patterns of soil dissolved inorganic nitrogen (DIN) in greenhouse vegetable systems, (3) to assess the impacts on soil properties and microbial community composition.

## 2 Materials and methods

### 2.1 Experimental sites and plant materials

The experiment was conducted from July 18 to September 25, 2024, in a greenhouse located in Lanling County, Shandong Province, China (34 °52′N, 117 °56′E; elevation 44 m). The experimental timeline included three key stages: before greenhouse solarization (July 20), after greenhouse solarization (August 20), and post-transplantation (September 20). The greenhouse solarization period lasted from July 20 to August 10. After August 10, the plastic film and greenhouse covering used for greenhouse solarization were removed to allow ventilation. Organic fertilizer was applied on August 20, followed by cucumber transplantation on August 25. September 24 marked 30 days after transplantation. Grafted cucumber was used as the experimental crop, with rootstock variety Zhenxinxin 431 (RSM NO.431) and scion variety Boxin 10-2. The planting density was 3,700 plants per mu (approximately 55,500 plants per hectare), using a wide-narrow row planting method with row spacing of 80 cm and 40 cm, and a plant spacing of 30 cm. Drip irrigation was used, and fruit harvesting was carried out during the cucumber harvesting stage.

### 2.2 Experimental design and field management

The experiment was conducted using a randomized complete block design with four treatments based on whether greenhouse solarization was applied and the type of organic amendment used after disinfection, with four replicates per treatment. Nutrient input levels for each treatment are detailed in Supplementary Table S1. The treatments included: (1) Only greenhouse solarization (CK), where no calcium cyanamide disinfection was applied and 33.3 t·ha^−1^ of chicken manure was used as basal fertilizer; (2) Famers’ Practice (FP), with calcium cyanamide applied via irrigation before greenhouse solarization and organic fertilizer input identical to CK; (3) bio-organic fertilizer treatment (BF), which involved greenhouse solarization as in FP but replaced chicken manure with 6 t·ha^−1^ of bio-organic fertilizer and 750 kg·ha^−1^ of kaolin clay; and (4) organic material treatment (OM), where greenhouse solarization was also conducted as in FP, with organic materials including 6.3 t·ha^−1^ biochar, 2.4 t·ha^−1^ peat, and 1.5 t·ha^−1^ fungal residue, and soil conditioner applied as in BF. The calcium cyanamide, sourced from Shandong Jiuzhouyuan Biotechnology Co., Ltd., was applied at 1,410 kg·ha^−1^in FP, BF, and OM. Before greenhouse solarization, diluted calcium cyanamide was applied through drip irrigation at 120 mm to the FP, BF, and OM plots. Following irrigation, the soil was covered with plastic film and the greenhouse was sealed to maintain high temperature conditions for 20 days.

### 2.3 Sample collection

#### 2.3.1 Cucumber sample collection

During the harvest period, cucumbers were harvested once they reached marketable size, with harvesting performed every 2–5 days. At each harvest, two uniformly growing cucumber plants were randomly selected from 54 plants located within the middle two rows of each plot. The plants were separated into roots, stems, leaves, and fruits for subsequent measurements.

#### 2.3.2 Soil sample collection

Soil samples were collected after transplantation. Three points were selected within each plot, and soil was sampled at 20 cm intervals from 0 to 200 cm depth. The three parallel samples from each depth were combined to form a composite sample. Fresh soil samples were sieved through a 2 mm mesh and stored at temperatures below 4.4 °C. Additionally, soil samples from the 0–60 cm layer (collected every 20 cm) were also taken, air-dried, and sieved through a 2 mm mesh for the determination of basic soil properties. For microbial community analysis, soil samples from the 0–20 cm layer were collected after transplantation by mixing three points per plot into one composite sample, sieved through 2 mm mesh, stored in ice boxes during transport, and subsequently preserved at −80 °C in the laboratory.

### 2.4 Sample measurements

#### 2.4.1 Cucumber yield, dry weight, nitrogen uptake, and K, Ca, Mg nutrient uptake

Total yield was calculated as the cumulative weight of harvested cucumbers during the harvesting period. Plant samples were washed with tap water, then dried to constant weight in a vacuum oven at 75 °C before weighing. Dry matter weight of fruits was determined by drying clean fruits in a 75 °C vacuum oven until constant weight. Samples were digested using the H₂SO₄-H₂O₂ method, and total nitrogen concentration was determined by the Kjeldahl method. Plant nitrogen uptake was calculated as the sum of nitrogen contents in roots, stems, leaves, and fruits, with nitrogen content of each organ obtained by multiplying dry weight by nitrogen concentration.

Plant samples separated into roots, stems, leaves, and fruits were ground and digested using microwave digestion with HNO₃-H₂O₂ (6 mL HNO₃ and 2 mL H₂O₂) in a microwave digestion system (CEM, Matthews, NC, United States). Potassium, calcium, and magnesium concentrations were measured using inductively coupled plasma optical emission spectrometry (ICP-OES, OPTIMA 3300 DV, Perkin-Elmer, United States). Quality control was conducted by adding standard reference material IPE126 (Wageningen University, The Netherlands). Potassium, calcium, and magnesium uptake by the plant was calculated as the sum of each nutrient in roots, stems, leaves, and fruits, with uptake per organ obtained by multiplying dry weight by nutrient concentration. Yield, dry weight, nitrogen uptake, and K, Ca, Mg nutrient uptake measurements were performed in three biological replicates.

#### 2.4.2 Soil DIN and basic properties measurement

Soil samples were extracted with 0.01 M CaCl₂, and concentrations of NH₄^+^ and NO₃^−^ were measured using a continuous flow analyzer (TRAACS 2000; Bran+Luebbe, Norderstedt, Germany). Soil organic carbon and total nitrogen were determined using a carbon-nitrogen analyzer (Elementar, Langenselbold, Germany). Available phosphorus was extracted with 0.5 mol L^−1^ NaHCO₃ (pH 8.5) and measured by the molybdenum-antimony anti-colorimetric method after filtration. Soil pH was measured using a pH meter with a soil-to-water ratio of 1:2.5. Available potassium, calcium, and magnesium were extracted with ammonium acetate and analyzed by ICP-OES (OPTIMA 7300 DV, Perkin-Elmer, United States). Measurements of soil DIN and basic properties were performed with four biological replicates.

#### 2.4.3 Soil bacterial DNA extraction and analysis

Soil genomic DNA was extracted using the CTAB method. DNA purity and concentration were assessed, and samples were diluted with sterile water to 1 ng/μl. The V3-V4 variable regions of 16S rRNA genes were PCR-amplified. Equal amounts of PCR products were pooled based on concentration. Target bands were purified using the Universal DNA Purification Kit (TianGen, China). Libraries were constructed using the NEBNext® Ultra DNA Library Prep Kit, quality-checked on an Agilent 5400, quantified by qPCR, and sequenced on the NovaSeq 6000 platform. Four biological replicates were sequenced.

Raw FASTQ sequences were imported using the QIIME tools import plugin for subsequent processing with QIIME2. Quality control, trimming, denoising, merging, and chimera removal were performed with the QIIME2 dada2 plugin (Callahan et al., 2016; Dubois et al., 2022). ANOVA was applied to identify bacterial taxa with differential abundance between groups and samples (Mandal et al., 2015). Alpha diversity indices at the feature sequence level and beta diversity indices including Bray-Curtis were calculated with QIIME2 core-diversity plugins to assess microbial community structure differences, which were visualized by PCA. PLS-DA was used to reveal relationships between microbial communities and sample categories. Redundancy analysis (RDA) and canonical correspondence analysis (CCA) were performed to explore potential correlations between microbial communities and environmental factors (Warton et al., 2011). Microbial functional profiles were predicted using PICRUSt2 software (Yang et al., 2023).

### 2.5 Statistical analysis

Statistical analyses were performed using IBM SPSS Statistics 24.0. One-way analysis of variance (ANOVA) was conducted to assess the effects of different treatments, with significance set at *p* < 0.05. Heatmaps were generated using Chiplot, while bar charts were created with GraphPad Prism (v8.0.2). Network diagrams were constructed using Cytoscape (v3.8.0) (Smoot et al., 2011), and other visualizations were produced using R software (v4.4.1) and its packages.

## 3 Results

### 3.1 Effects of different nutrient management practices on the growth of greenhouse cucumber

**Figure 1:**
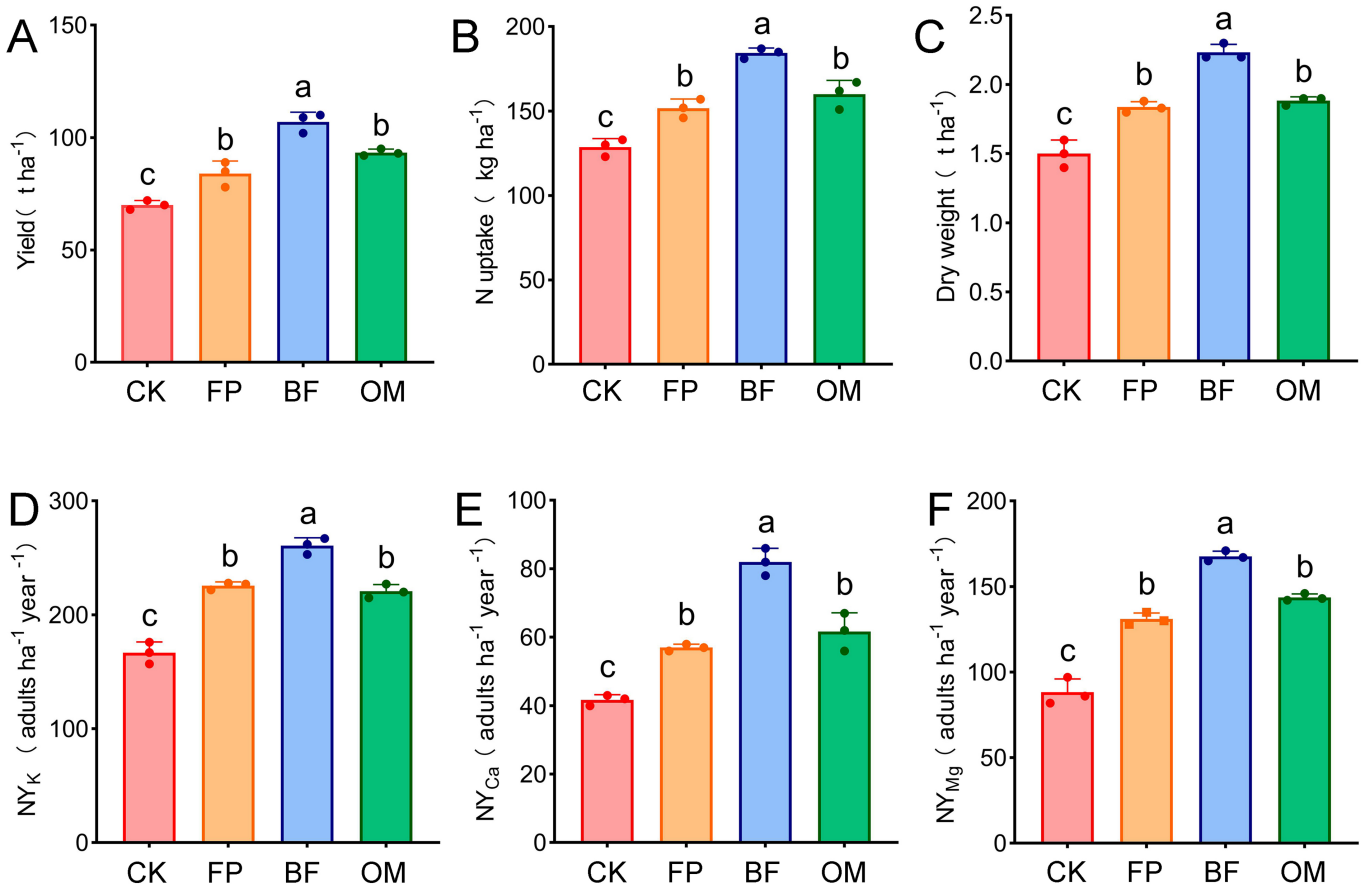
Effect of different nutrient management practices on the growth of facility cucumber. **(A)** Cucumber yield. **(B)** Cucumber nitrogen uptake. **(C)** Cucumber fruit dry weight. **(D)** Cucumber potassium nutrient yield. **(E)** Cucumber calcium nutrient yield. **(F)** Cucumber magnesium nutrient yield. The letters (a, b, c, d) represent the results of independent samples *t*-tests for statistical significance. Different letters indicate significant differences between treatments, while the same letter indicates no significant difference.

To investigate the effects of FP, BF, and OM treatments on greenhouse cucumber, yield, nitrogen uptake, dry weight, and nutrient uptake of potassium, calcium, and magnesium were measured (Figure 1). The results showed that compared to the control, yields in FP, BF, and OM treatments increased by 1.20, 1.53, and 1.33 times, respectively. The yield under BF treatment was significantly higher than that of CK, FP, and OM treatments, while no significant difference was observed between FP and OM (Figure 1A). Regarding fruit dry weight, FP, BF, and OM increased it by 22.7, 48.7, and 25.3%, respectively, compared to CK (Figure 1C), which showed a significant increase under BF treatment. In terms of nutrient quality, nitrogen uptake was highest in the BF treatment at 184.3 kg·ha^−1^, whereas no significant differences were found between FP (151.7) and OM (160.2) (Figure 1B). Furthermore, nutrient yield, which integrates crop yield, nutrient concentration in fruits, and dietary recommendations, is an important indicator for evaluating crop quality. Compared to the CK group, FP, BF, and OM significantly increased the nutritional yield of potassium, calcium, and magnesium in cucumbers, with BF showing the optimal nutritional yield. There were significant differences between BF and both FP and OM, while no significant difference was observed between FP and OM (Figures 1D–F). In summary, different fertilizer treatments effectively improved yield, fruit dry weight, and nutrient yield of greenhouse cucumber, with BF showing the best performance.

### 3.2 Effects of different nutrient management practices on soil DIN migration

**Figure 2:**
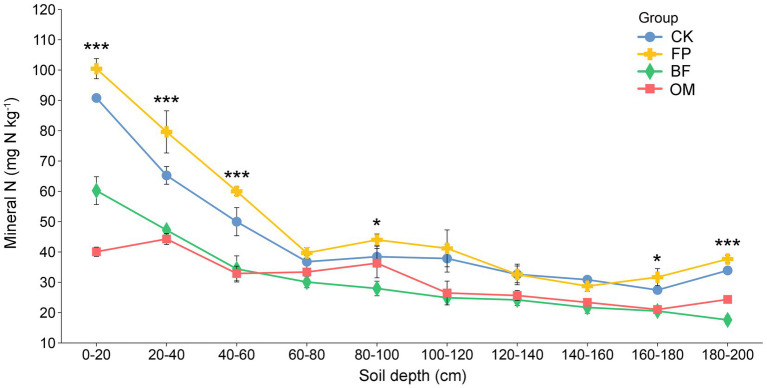
Changes in soil dissolved inorganic nitrogen (DIN) content (0–200 cm) under different nutrient management practices. Differences among treatments were evaluated using ANOVA. Significance levels: *p < 0.05; **p < 0.01; ***p < 0.001.

Soil DIN, including ammonium nitrogen (NH₄^+^) and nitrate nitrogen (NO₃^−^), not only influences nutrient supply for cucumber but also affects the soil environment. The content of DIN in the soil profile from 0 to 200 cm under different treatments showed an overall decreasing trend across all four treatment groups. Notably, the CK and FP treatments exhibited significantly higher DIN contents in the 0–60 cm soil layer compared to BF and OM, with this difference being especially pronounced in the 0–20 cm layer (90.8, 100.4, 60.2, and 40.1 mg N kg^−1^, respectively) (Figure 2). As soil depth increased, DIN content in BF and OM treatments remained lower than that in CK and FP. The lowest value was observed in BF at 180–200 cm depth, with 17.6 mg N kg^−1^. These results indicate that CK and FP treatments led to nitrogen accumulation in the topsoil, promoting varying degrees of DIN migration to deeper soil layers and resulting in nitrogen leaching losses. Overall, BF and OM treatments effectively mitigated nitrogen loss while maintaining cucumber yield and quality, with BF showing relatively better performance.

### 3.3 Effects of different nutrient management practices on basic soil properties

**Figure 3:**
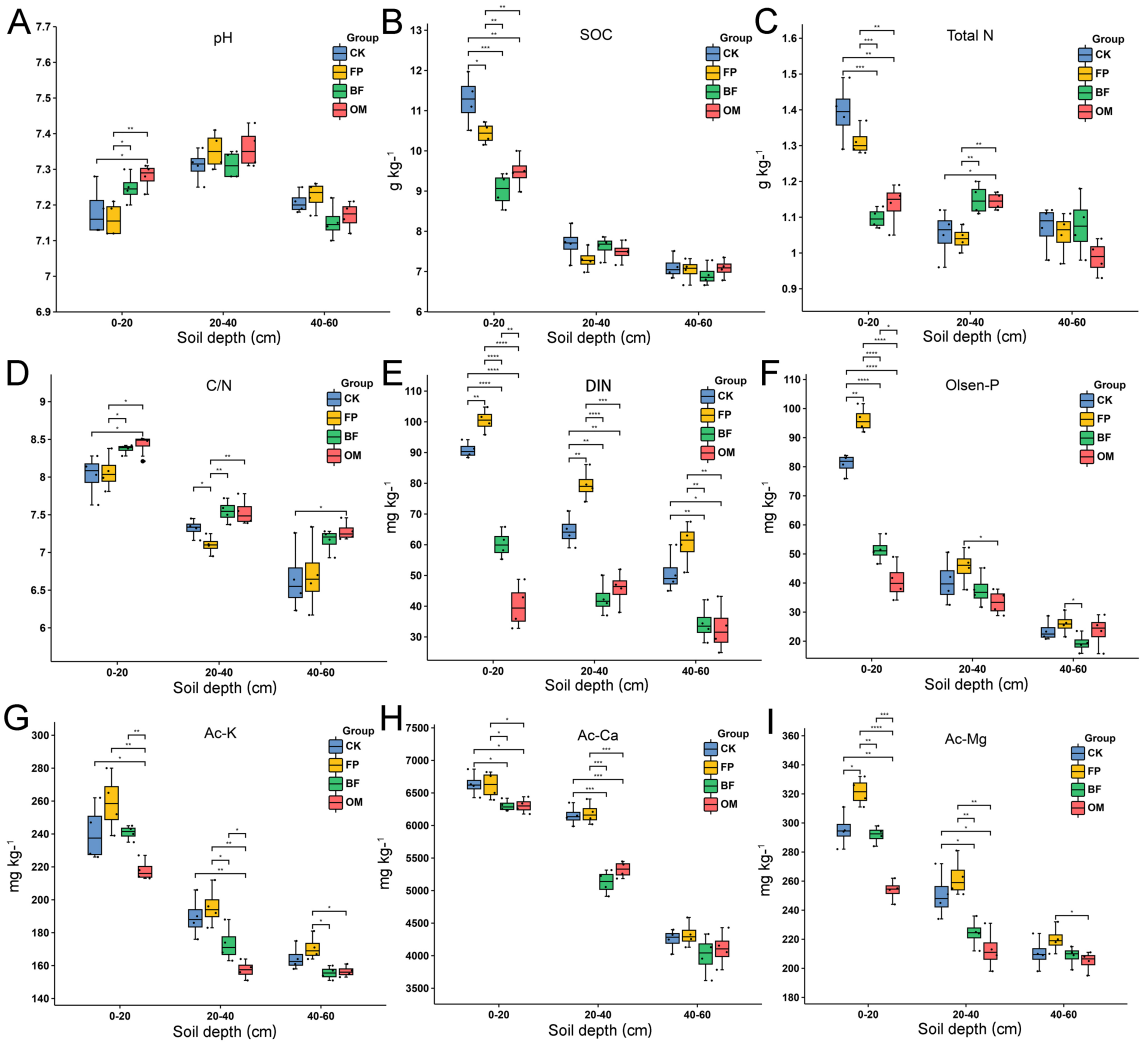
Basic properties of 0–60 cm soil under different nutrient management practices. **(A)** pH. **(B)** Soil organic carbon. **(C)** Total nitrogen. **(D)** Carbon to nitrogen ratio. **(E)** Dissolved inorganic nitrogen. **(F)** Olsen phosphorus. **(G)** Available potassium. **(H)** Available calcium. **(I)** Available magnesium. Differences among treatments were evaluated using ANOVA. Significance levels: *p < 0.05; **p < 0.01; ***p < 0.001.

Given the significant changes in DIN observed within the 0–60 cm soil layer, we further examined the variations in basic soil properties (Figure 3). Except for available calcium, all measured parameters-including pH, soil organic carbon (SOC), total nitrogen (TN), C/N ratio, Olsen-P, available potassium (Ac-K), and available magnesium (Ac-Mg), showed significant differences in the 0–20 cm soil layer. BF and OM significantly increased soil pH at 0–20 cm (Figure 3A), with no notable differences at deeper layers. For SOC, the FP, BF and OM treatment significantly decreased its content in the 0–20 cm layer compared with CK (Figure 3B). Although TN content in FP was higher than that in BF and OM at 0–20 cm (Figure 3C), the C/N ratio was significantly higher in BF and OM compared to FP and CK (Figure 3D). For DIN in the 0–60 cm layer, the highest values were found in FP, followed by CK, BF, and OM (Figure 3E). Similar to DIN, available phosphorus (Olsen-P) was significantly lower in BF and OM compared to CK and FP at 0–20 cm (Figure 3F). Ac-K content was highest in FP across the 0–40 cm layer, with significantly lower values in BF and OM (Figure 3G). Available calcium (Ac-Ca) showed significant differences only at 0–40 cm, where FP had the highest levels, followed by CK (Figure 3H). For Ac-Mg, FP consistently exhibited the highest content across the 0–60 cm layer, while OM showed the lowest (Figure 3I). In summary, the FP treatment led to the accumulation of most soil nutrients in the topsoil (0–20 cm), whereas BF and OM effectively reduced their excessive accumulation and potential waste.

### 3.4 Effects of different nutrient management practices on soil microbial communities

**Figure 4:**
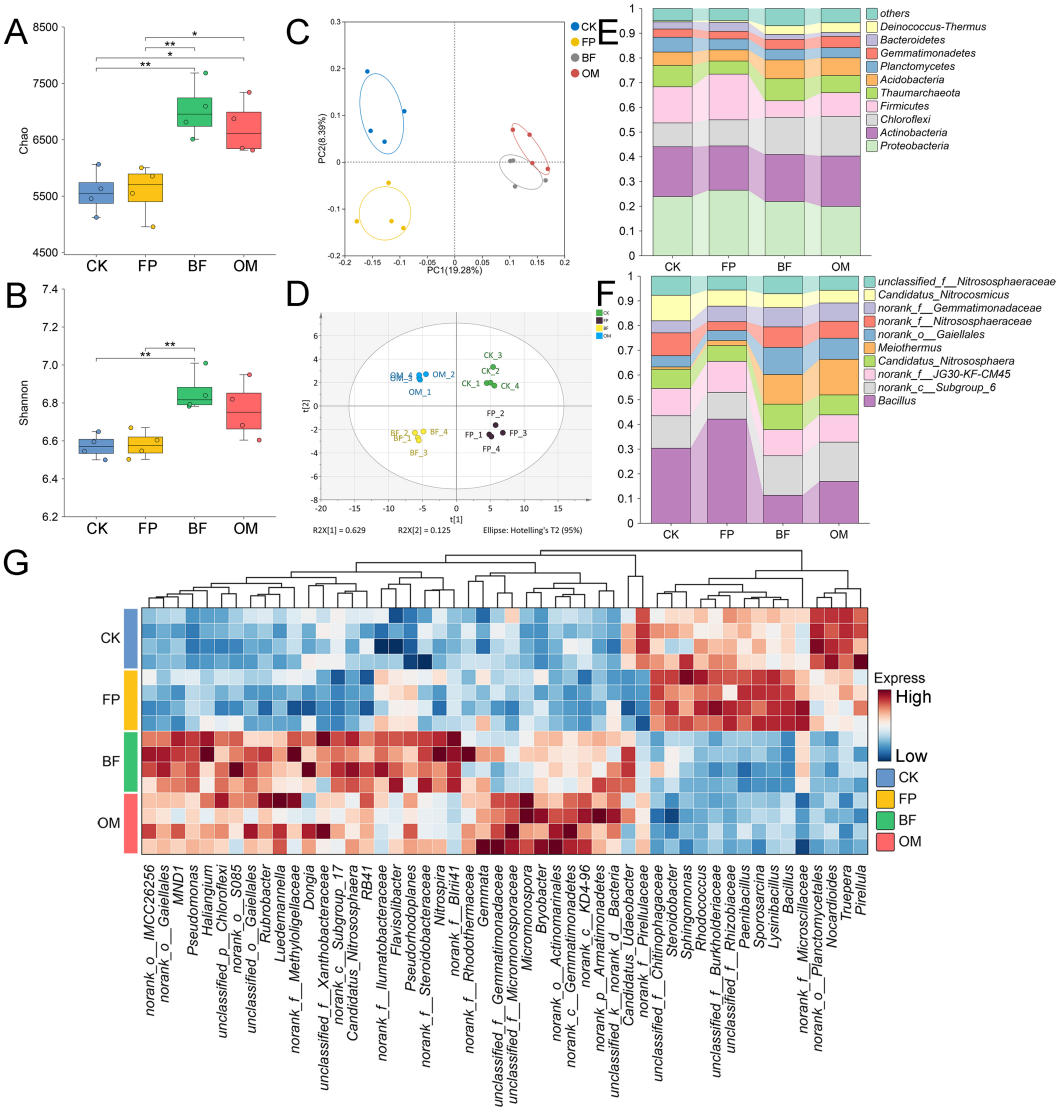
Characterization of bacterial communities under different nutrient management practices. **(A)** Chao index. **(B)** Shannon index. **(C)** PCA analysis. **(D)** PLS-DA analysis. **(E)** Bacterial community composition at the phylum level. **(F)** Bacterial community composition at the genus level. **(G)** Heatmap of bacterial abundance in the top 50 bacteria of abundance.

Microbial activity is closely linked to crop performance and environmental conditions. To evaluate the impact of fertilization treatments on microbial community dynamics, 16S rRNA sequencing was performed on soil bacterial communities from the 0–20 cm layer (Figure 4). Based on a 97% OTU sequence similarity threshold, a total of 983,771 high-quality sequences were obtained. The bacterial communities included 47 phyla, 123 classes, 357 orders, 617 families, and 1,227 genera. The bacterial Shannon rarefaction curves plateaued toward the end, indicating sufficient sequencing depth (Supplementary Figures S1A,B), and the coverage of all samples was close to 1. In terms of α-diversity, both Chao and Shannon indices revealed that bacterial diversity was highest under the BF treatment, followed by OM (Figures 4A,B), indicating that optimized fertilizer applications significantly enhanced soil bacterial diversity. Principal component analysis (PCA) further revealed clear separations among CK, FP, and BF/OM treatments (Figure 4C), with BF and OM clustering closely together, suggesting significant differences in bacterial communities between optimized and conventional treatments. PLS-DA analysis supported this finding, demonstrating that fertilization management effectively altered microbial community structures (Figure 4D).

At the phylum level, Proteobacteria, Actinobacteria, and Chloroflexi were dominant taxa, accounting for more than 50% of the total relative abundance (Figure 4E). At the genus level, the abundance of *Bacillus* was significantly reduced in both BF and OM treatments, with the greatest decrease observed in OM (Figure 4F). In contrast, the relative abundance of *norank_c_Subgroup_6*, *Candidatus_Nitrososphaera*, *Meiothermus*, and *norank_o_Gaiellales* markedly increased in BF, by 47.8, 62.8, 477.1, and 173%, respectively, compared to FP, becoming dominant genera. Cluster analysis of the top 50 most abundant genera revealed two distinct groups: one dominated by BF and OM, and the other by CK and FP, further confirming that nutrient management practices significantly altered the composition of the soil microbial community.

### 3.5 Identification of indicator microorganisms under different nutrient management practices

**Figure 5:**
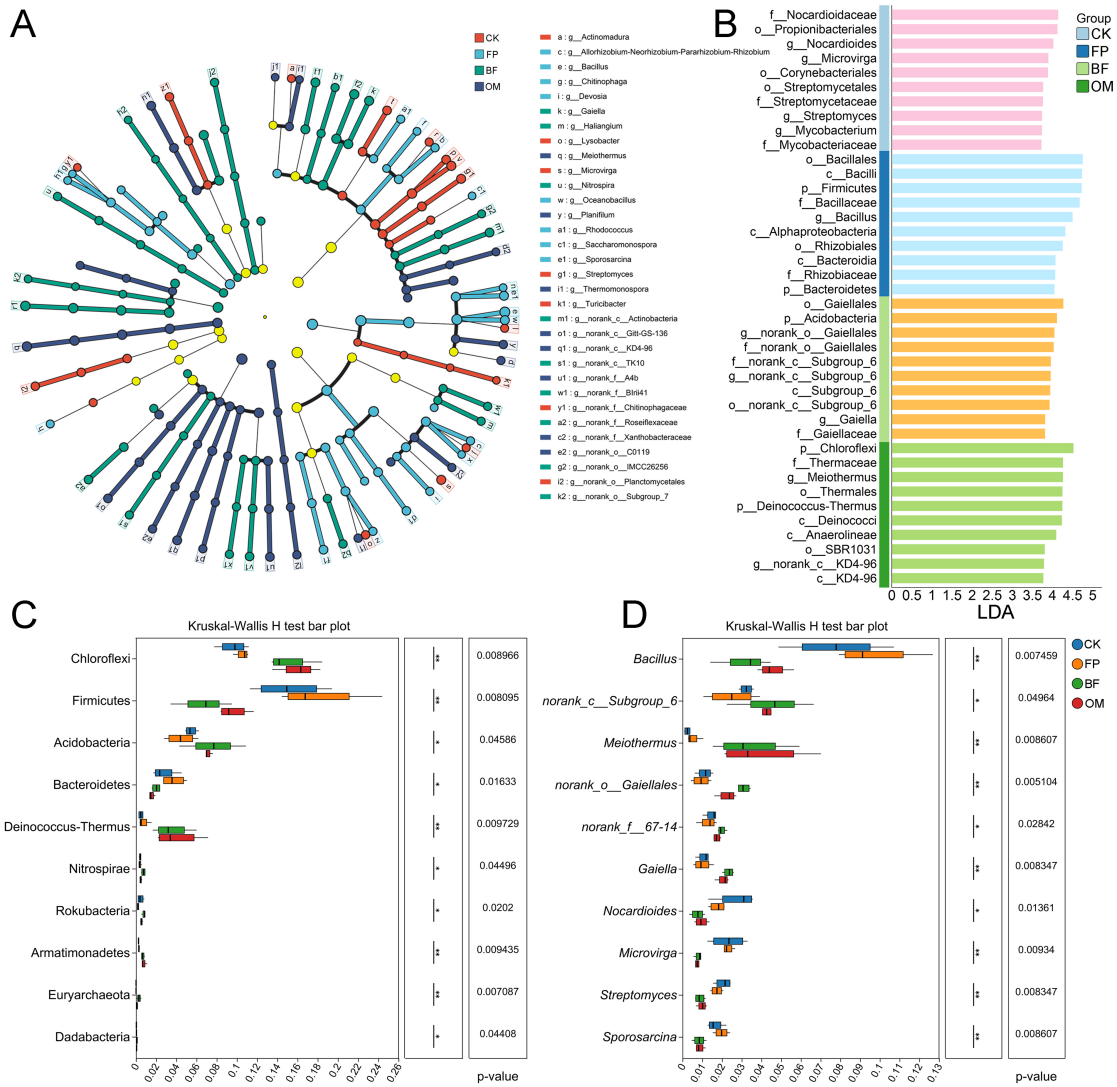
Screening of microorganisms characterized by different nutrient management practices. **(A)** LEfSe multilevel species hierarchical tree diagram. **(B)** LDA discrimination results. **(C)** Phylum level difference test. **(D)** Genus level difference test. Differences among treatments were evaluated using ANOVA. Significance levels: *p < 0.05; **p < 0.01; ***p < 0.001.

To further investigate microbial community differences among treatments, LEfSe was employed to identify characteristic bacterial taxa associated with each fertilization regime (Figure 5A). In the CK group, *Actinomadura*, *Lysobacter*, *Microvirga*, and *Streptomyces* were identified as characteristic genera (Figure 5A). At the family level, Nocardioidaceae and the order Streptomycetales had the greatest impact on group differentiation, with LDA scores of 4.14 and 3.76, respectively (Figure 5B). In the FP group, *Bacillus*, *Chitinophaga*, and *Devosia* were the dominant genera, with enrichment further supported by the class Bacilli and the order Bacillales (Figure 5B).

In the BF treatment, *norank_c__Subgroup_6* and *norank_o__Gaiellales* were identified as key indicator taxa, with notable enrichment of the corresponding families (f_norank_c__Gaiellales, LDA = 4.03; f_norank_c__Subgroup_6, LDA = 3.96). For OM, *Meiothermus* was identified as the dominant indicator microorganism, with the highest LDA score (4.26), suggesting a strong influence on community structure. To visually assess the relative abundance of indicator taxa across treatments, Kruskal-Wallis rank sum tests were performed at the phylum and genus levels (Figure 5C). At the phylum level, Firmicutes were significantly enriched in the FP group (*p* = 0.008), while Acidobacteria and Chloroflexi were dominant in BF (*p* = 0.040) and OM (*p* = 0.008), respectively. At the genus level, *Bacillus* (*p* = 0.007) and *Sporosarcina* (*p* = 0.008) were significantly more abundant in FP, whereas *norank_c__Subgroup_6* (*p* = 0.049), *Gaiella* (*p* = 0.008), and *Meiothermus* (*p* = 0.008) were dominant in BF and OM (Figure 5D).

### 3.6 Integrated analysis of soil properties and microbial communities under different nutrient management practices

**Figure 6:**
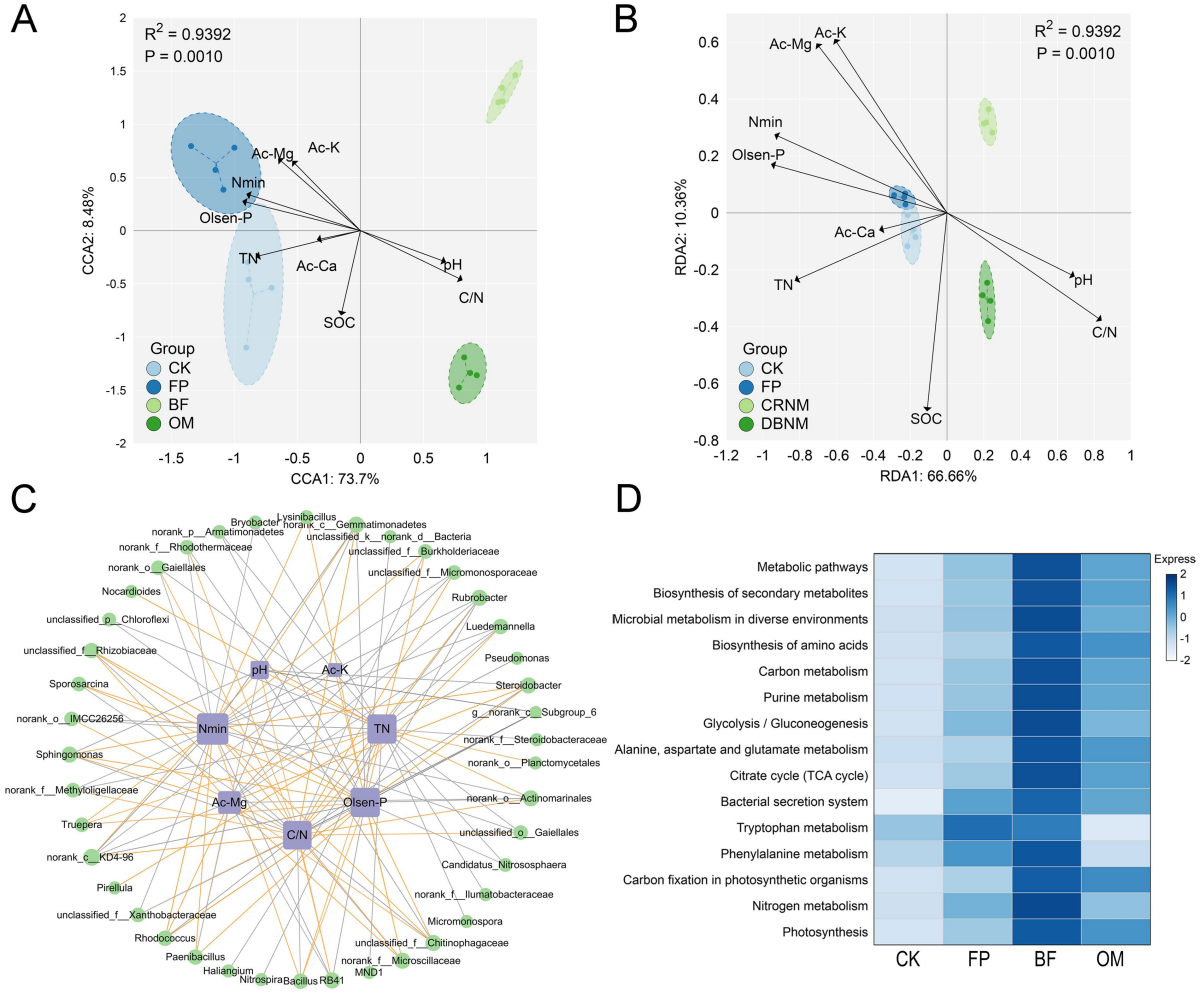
Integrated analysis of microbial communities and soil physicochemical properties. **(A)** CCA analysis. **(B)** RDA analysis. **(C)** Correlation analysis. **(D)** KEGG functional enrichment analysis.

Redundancy analysis (RDA), canonical correspondence analysis (CCA), and correlation analysis were performed to evaluate the relationships between soil properties and microbial communities in the 0–20 cm layer under each treatment (Figure 6). In the CCA plot (Figure 6A), available Mg (Ac-Mg), available K (Ac-K), available Ca (Ac-Ca), mineral nitrogen (Nmin), Olsen-P, and total nitrogen (TN) exerted strong positive influences on the microbial communities of the CK and FP treatments, with most of these soil properties showing the highest correlations with FP. Soil organic carbon (SOC) was not significantly correlated with any treatment, whereas pH and the C/N ratio were more closely associated with OM. RDA yielded a pattern consistent with CCA (Figure 6B), confirming that differences in soil properties induced by the various treatments shaped microbial community composition.

Correlation analysis (Figure 6C) revealed that *Sphingomonas* was strongly negatively correlated with pH (*r* = −0.85), while *norank_c__Subgroup_6* was positively correlated with pH (*r* = 0.64). With respect to TN, significant correlations were observed for *Bacillus* (*r* = 0.72), *Gaiella* (*r* = −0.81), *Candidatus Nitrososphaera* (*r* = −0.74), and *Pseudomonas* (*r* = −0.89). The C/N ratio showed significant negative correlations with *Bacillus* (*r* = −0.71) and *Sphingomonas* (*r* = −0.78), whereas significant positive correlations were found with *norank_c__Subgroup_6* (*r* = 0.64), *Gaiella* (*r* = 0.72), and *Candidatus Nitrososphaera* (*r* = 0.66). Similar trends were detected for mineral nitrogen and Olsen-P, both were positively correlated with *Bacillus* and *Pseudomonas* (*r* = 0.74–0.86) and negatively correlated with *Gaiella*, *norank_c__Subgroup_6*, and *Candidatus Nitrososphaera*. Collectively, the RDA/CCA and correlation analyses indicate that the BF treatment effectively mitigated soil nutrient losses and identified soil properties that drive microbial community composition.

Functional prediction of the bacterial communities (Figure 6D) showed that BF was enriched in pathways related to Metabolic pathways, Microbial metabolism in diverse environments, Phenylalanine metabolism, Nitrogen metabolism, Citrate cycle, and Carbon metabolism. These functions may enhance the transformation and utilization of carbon and nitrogen, thereby facilitating cucumber nutrient uptake and reducing residual soil nutrients.

## 4 Discussion

### 4.1 Response of cucumber growth performance to nutrient management strategies

A typical characteristic of protected vegetable production systems in China is their high cropping index, excessive fertilizer input, and severe leaching losses, all of which place significant pressure on environmental protection. In this study, optimizing fertilizer input ratios effectively improved cucumber yield. Compared to the CK treatment, the BF treatment increased yield by 53% and improved dry weight by 48.7%. This may be related to soil-borne diseases caused by excessive fertilizer application. Excessive fertilization not only exacerbates soil acidification but also creates favorable conditions for the occurrence of diseases (Wei et al., 2024; Zhang et al., 2022). In the BF and OM treatments, the incorporation of kaolinite and organic materials helped restore soil quality by reducing the input of excessive manure-based nitrogen, thereby mitigating the risk of soil-borne diseases. In addition, the study found that the optimized integrated management strategy of organic and inorganic fertilizers has a positive impact on cucumber growth. The use of slow-release organic fertilizers has been shown to enhance soil DIN concentrations and improve both grain yield and nitrogen use efficiency in rice production (Wang Y. et al., 2025). Mercer also demonstrated that under moderate irrigation and nitrogen inputs, cotton yield as well as nitrogen and water use efficiency were maximized without the need for excessive resource input (Baird et al., 2024). Furthermore, in the BF treatment, the application of commercial animal-derived organic fertilizer may have promoted nitrogen uptake in cucumber, thereby increasing yield. This effect could be attributed to enhanced microbial transformation processes and the presence of bioactive compounds (Quan et al., 2015; Chaichi et al., 2017). This study also introduced the concept of nutritional yield to evaluate the effects of different nutrient management strategies on the uptake of potassium, calcium, and magnesium in cucumber. The BF treatment significantly outperformed FP in terms of nutritional yield. By modifying the type and dosage of organic fertilizers and adding high-pH soil amendments rich in calcium, magnesium, and silicon, cucumber growth was promoted and nutrient uptake of Ca and Mg was enhanced.

### 4.2 Effects on soil physicochemical properties and DIN migration

In the present study, we found that the application of organic fertilizers significantly increased the concentrations of soil DIN, available phosphorus, and exchangeable potassium in surface soil. Moreover, compared to the FP treatment, the addition of bio-organic fertilizers and organic materials significantly enhanced the carbon-to-nitrogen (C/N) ratio in the topsoil, which is likely attributable to the type and amount of organic inputs. Previous studies have reported that under conventional fertilization regimes, large applications of organic manure lead to nutrient accumulation in greenhouse vegetable soils, consistent with our findings (Bai et al., 2020).

On one hand, the application of organic fertilizers increases total nutrient input, which may promote the downward movement of nitrogen. On the other hand, some studies suggest that the characteristics of organic fertilizers can also reduce nitrogen leaching losses (Tei et al., 2020; Xu et al., 2020). Our results indicate that the FP treatment led to significantly higher DIN concentrations in the 0–60 cm soil profile than treatments with bio-organic fertilizers or organic materials. During the early growth stages of cucumber, root systems are not yet fully developed, making it difficult for roots to access nitrogen below 20 cm.

### 4.3 Effects on soil microbial communities

Soil microorganisms interact closely with the rhizosphere environment of crops. Based on RDA/CCA analysis of bacterial community composition at the genus level and soil environmental factors, we observed strong associations between bacterial communities and soil physicochemical properties across different treatments. Previous studies have confirmed significant correlations between variations in soil nutrient content and changes in bacterial community structure, particularly with available potassium, total nitrogen, and total carbon (Han et al., 2025; Lin et al., 2025; Peng et al., 2025). In our study, post-transplantation soil nutrient status exerted a major influence on the structure of the bacterial community.

Specifically, *Actinomadura*, *Lysobacter*, *Microvirga*, and *Streptomyces* were identified as characteristic genera in the CK group, *Bacillus* was dominant in FP, *norank_c__Subgroup_6* and *Gaiella* were enriched in BF, while *Meiothermus* was predominant in OM. *Microvirga* and *Streptomyces* have been shown to be positively correlated with consecutive years of tobacco monocropping (Li et al., 2024), and are also considered deleterious microbes contributing to yield decline in soybean (Neupane et al., 2021). The presence of these genera in non-greenhouse solarization cucumber soils may further affect soil conditions and crop growth. *Norank_c__Subgroup_6* has been identified as a beneficial rhizosphere bacterium recruited by the halophyte Suaeda salsa to promote root development (Diao et al., 2024). Additionally, it has been implicated in antibiotic resistance regulation, serving as the only potential host of the sul2 gene in wheat rhizosphere soils (Guo et al., 2020). In the intercropping system of *Pteris vittata* L. and *Sedum alfredii Hance*, the application of mixed fertilizers increased the abundance of *norank_c__Subgroup_6*, contributing to the remediation of heavy metal-contaminated soils (Yang et al., 2025).

Regarding *Gaiella*, its relative abundance was increased by the addition of maize root exudates (from 2.7 to 4.7%), and was positively correlated with shoot phosphorus concentration and soil phosphatase activity, suggesting its potential role in phosphorus mobilization (Wang G. et al., 2025). *Meiothermus* was found to increase by 8.75% in relative abundance during summer fallow when soil solarization was combined with manure amendment, indicating its contribution to improved soil quality (Wang et al., 2023). The abundance changes of these microorganisms under different treatments mainly depend on the type of fertilizer, application ratio, and management practices. In summary, the BF treatment significantly enhanced soil bacterial diversity, increased the abundance of beneficial bacteria, and altered the microbial community structure.

## 5 Conclusion

This study investigated the effects of different nutrient management strategies on cucumber growth, soil nutrients, and microbial communities. The results identified that BF treatment outperformed other treatments in enhancing cucumber yield, dry weight, and nutrient uptake, particularly in terms of potassium, calcium, and magnesium nutritional yield. Additionally, BF significantly reduced the migration of inorganic nitrogen in deeper soil layers while also decreasing nutrient accumulation in the 0–20 cm soil depth. The optimization measures also altered the soil microbial community composition. These findings suggest that optimizing nutrient management practices can improve crop productivity, reduce nutrient losses, and promote more sustainable agricultural practices.

## Data Availability

The original contributions presented in the study are included in the article/Supplementary material, further inquiries can be directed to the corresponding author.
