# Supplementary figures and images for: Soil disinfestation and optimized nutrient management reduces nitrogen leaching and shapes soil microbial community composition in greenhouse cucumber production systems
Source: Front Microbiol. 2025 Sep 5;16:1663041. doi: 10.3389/fmicb.2025.1663041 (PMC12446332; doi:10.3389/fmicb.2025.1663041)

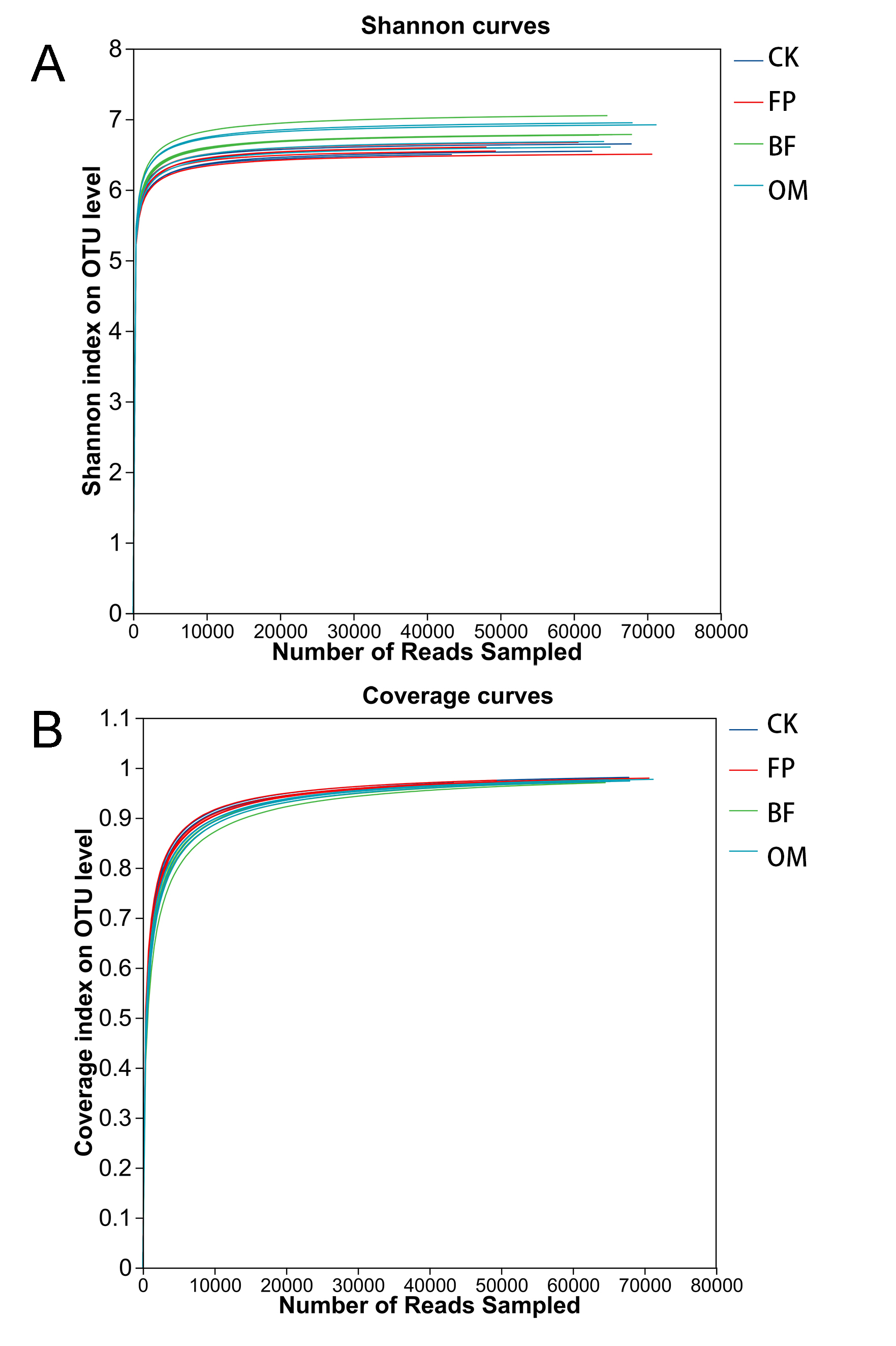

Supplement: Supplementary file 1 [file Image_1.JPEG]
